# A Systematic Review of Surgical Techniques for the Repair of Capitellar Fractures

**DOI:** 10.7759/cureus.81304

**Published:** 2025-03-27

**Authors:** Kashif Memon, Manahil Awan, Lara Alsadoun, Shahzad Ahmad, Samuel Chan, Socrates Kalogrianitis, Arslan A Abro

**Affiliations:** 1 Trauma and Orthopedics, Queen Elizabeth Hospital Birmingham, Birmingham, GBR; 2 Trauma and Orthopedics, Chelsea and Westminster Hospital, London, GBR; 3 Cardiac Surgery, Queen Elizabeth Hospital Birmingham, Birmingham, GBR; 4 Orthopedics, Queen Elizabeth Hospital Birmingham, Birmingham, GBR

**Keywords:** capitellum, fixation, fracture, outcome studies, patient outcomes, surgical approach

## Abstract

The rarity of capitellum fractures makes them a thorny problem in orthopedic practice, as fracture reduction and repair to restore joint function within a complex elbow joint are difficult. Properly treated, these fractures can avoid complications such as stiffness, instability, and posttraumatic arthritis. Several surgical techniques optimize patient outcomes, including open reduction with internal fixation using Herbert screws, buttress plating, and headless compression screws (HCS). The choice of technique, however, is determined by many factors, including fracture type, patient characteristics, and surgeon preference. This systematic review compares the clinical and functional outcomes of surgical techniques for capitellar fracture repair. Factors influencing the selection of surgical approach are also reviewed, including fracture complexity, patient demographics, and bone quality. To perform a systematic literature search, we followed Preferred Reporting Items for Systematic Reviews and Meta-Analyses (PRISMA) guidelines and searched the major databases, including PubMed, Cochrane Library, Embase, and Web of Science. Search terms included "capitellar fracture", "elbow fracture", and "surgical fixation techniques". Clinical or biomechanical outcomes of capitellar fracture fixation using different surgical techniques were included, including fixation stability, range of motion, healing rates, and complication rates. In detail, three foundational studies were analyzed in depth, with the use of Herbert screws, Kirschner wires, and buttress plating. Clinical trials suggest that Herbert screws enable earlier mobilization and improved functional outcomes, particularly in younger patients with Type I fractures. Biomechanical studies, such as those using HCS with buttress plating, indicate enhanced stability, especially in osteoporotic bone conditions. Factors such as patient age, bone quality, and fracture pattern appear to influence the choice of surgical technique. Herbert screws provide effective fixation and support early mobilization, making them suitable for stable fractures in healthier patients. However, in cases of osteoporotic or complex fractures, augmented techniques, such as buttress plating, may be more appropriate to improve stability and reduce the risk of fixation failure. The selection of surgical techniques for capitellar fractures should take into account patient-specific factors to optimize clinical outcomes, and this review emphasizes the need for a tailored approach in selecting surgical techniques for capitellar fractures.

## Introduction and background

Capitellar fractures, while relatively uncommon, pose significant challenges in orthopedic practice due to the complexity of the elbow joint's anatomy and the intricacies of ensuring proper joint function after injury [1]. These fractures typically involve the capitellum, an essential structure of the distal humerus that articulates with the radial head, and are commonly observed in high-impact trauma or falls on an outstretched hand [2]. Accurate and effective surgical repair is crucial to restore elbow stability, range of motion, and overall limb function, as suboptimal fixation can lead to debilitating complications such as joint stiffness, instability, and posttraumatic arthritis [3]. In recent years, many surgical techniques have been developed, each aimed at improving patient outcomes, yet most techniques are selected based on such things as fracture type, patient age, comorbidity, and the surgeon’s expertise and preference.

A comparison of the surgical techniques available for capitellar fracture repair and the factors that influence the selection of these techniques is essential. Existing methods include open reduction with internal fixation using Herbert screws, buttress plating, and novel approaches that combine different surgical strategies [4]. Some techniques are biomechanically more stable and may be easier to fixate, and others may be advantages over certain types of fractures or for specific types of patients. A systematic review of the efficacy and safety of these various techniques, in conjunction with knowledge of the factors driving surgical decision-making, can yield useful information to optimize care for patients with capitellar fractures.

To ensure a rigorous approach to this review, we structured our analysis using the Population, Intervention, Comparison, Outcome, Study Design framework, which defines key elements essential to the scope of this research [5]. Population (P) refers to patients diagnosed with capitellar fractures, a demographic presenting unique challenges due to the fracture’s anatomical location and functional demands on the joint. Intervention (I) includes various surgical techniques employed in capitellar fracture repair, such as open reduction and internal fixation (ORIF; e.g., using Herbert screws or buttress plating). Finally, Comparison (C) compares these techniques against each other to determine which has the greatest impact in achieving optimal clinical outcomes. Outcome (O) measures include functional and clinical endpoints, including fracture healing, range of motion, joint stability, and complication rates (nonunion, joint stiffness, and infection). Study design (S) includes randomized controlled trials and clinical trials that produce empirical evidence and biomechanical insight into capitellar fracture repair.

This systematic review seeks to systematically review the literature on the surgical techniques used in capitellar fracture repair, focusing on clinical and functional outcomes. In addition, this review also aims to identify and analyze the factors influencing the selection of surgical techniques, including fracture type, patient characteristics, and surgeon preference. In this review, we synthesize available evidence to provide insights that may assist clinicians in choosing the most appropriate surgical approach for a given patient based on their unique needs and to enhance patient outcomes in capitellar fracture cases.

## Review

Methods

Search Strategy

To systematically and specifically search for capitellar fracture repair techniques, we used a combination of Medical Subject Headings and free-text keywords in major databases (PubMed, Cochrane Library, Embase, and Web of Science). The search terms used were directed both at the population and intervention components, using terms such as "capitellar fracture", "elbow fracture", and "distal humerus fracture" to represent the anatomy and fracture types of interest. Surgical fixation, open reduction, internal fixation, Herbert screws, Kirschner wires, buttress plating, and other intervention keywords were used to identify the most common approaches to capitellar fracture management. Terms were connected using Boolean operators (AND, OR) to search broadly enough to include a variety of studies but precisely enough to identify relevant findings in surgical approaches to capitellar fractures.

To further refine the search strategy, outcome-related terms were added, including "stability", "range of motion", "union rate", "complications", and "functional outcomes", thus prioritizing clinical and biomechanical outcomes. We also included filter criteria for randomized controlled trials, clinical trials, biomechanical studies, and systematic reviews where possible. By utilizing these specific terms and filters, we aimed to identify high-quality studies that compare surgical techniques and evaluate the efficacy of different fixation methods. This structured approach, aligned with the Preferred Reporting Items for Systematic reviews and Meta-Analyses guidelines [6], ensures that the review comprehensively addresses capitellar fracture repair, consolidating the best available evidence for clinical guidance.

Eligibility Criteria

Studies were considered eligible for inclusion in this systematic review if they met the following criteria: 1) the study was conducted in adults with capitellar fractures and surgical intervention as primary treatment, including ORIF via Herbert screws, Kirschner wires, and buttress plating; 2) clinical, functional, or biomechanical outcomes that have relevance to the comparative effectiveness of capitellar fracture repair techniques were reported; 3) studies were based on randomized controlled trial, clinical trial, retrospective study, or biomechanical evaluation design; and 4) studies that exclusively focused on nonsurgical management, fractures in pediatric populations, noncapitellar elbow fractures, case reports, and reviews lacking original clinical or biomechanical data were excluded. By applying these eligibility criteria, we aimed to compile high-quality evidence that reflects various surgical approaches' clinical and biomechanical outcomes, supporting a comprehensive assessment of optimal techniques for capitellar fracture repair.

Data Extraction

Data extraction was conducted systematically to ensure consistency and accuracy in capturing relevant study details. For each included study, key information was extracted, including authorship, publication year, study design, sample size, patient demographics, fracture type, surgical techniques used (e.g., Herbert screws, Kirschner wires, and buttress plating), comparison techniques, outcome measures, follow-up duration, primary findings, and noted limitations. Outcome data were further categorized into clinical and functional endpoints, such as a range of motion, joint stability, union rates, and complication incidence, as well as biomechanical results where applicable. Quantitative measures were recorded where possible to facilitate comparative analysis of fixation techniques across studies. Data extraction was independently verified by multiple reviewers to minimize error, with any discrepancies resolved through discussion to ensure the integrity of the extracted dataset.

Data Analysis and Synthesis

Data analysis and synthesis were conducted to compare clinical and functional outcomes across different surgical techniques for capitellar fracture repair. Studies were grouped based on the type of fixation method, including Herbert screws, Kirschner wires, and buttress plating, allowing for a structured comparative analysis. Outcome data, such as range of motion, union rates, joint stability, and complication frequencies, were synthesized to identify trends and differences between the techniques. Qualitative synthesis was applied to interpret biomechanical results, particularly in studies that included cadaveric models, to evaluate stability and load-bearing capacity. Where appropriate, descriptive statistics were used to summarize findings across studies, highlighting consistent trends and notable variations. Due to heterogeneity in study designs and outcome measures, a narrative synthesis approach was employed to integrate findings, emphasizing the clinical implications of each technique's strengths and limitations. This comprehensive synthesis provides insights into the effectiveness and suitability of each surgical method for capitellar fracture repair, guiding recommendations for clinical practice.

Results

Study Selection Process

**Figure 1:**
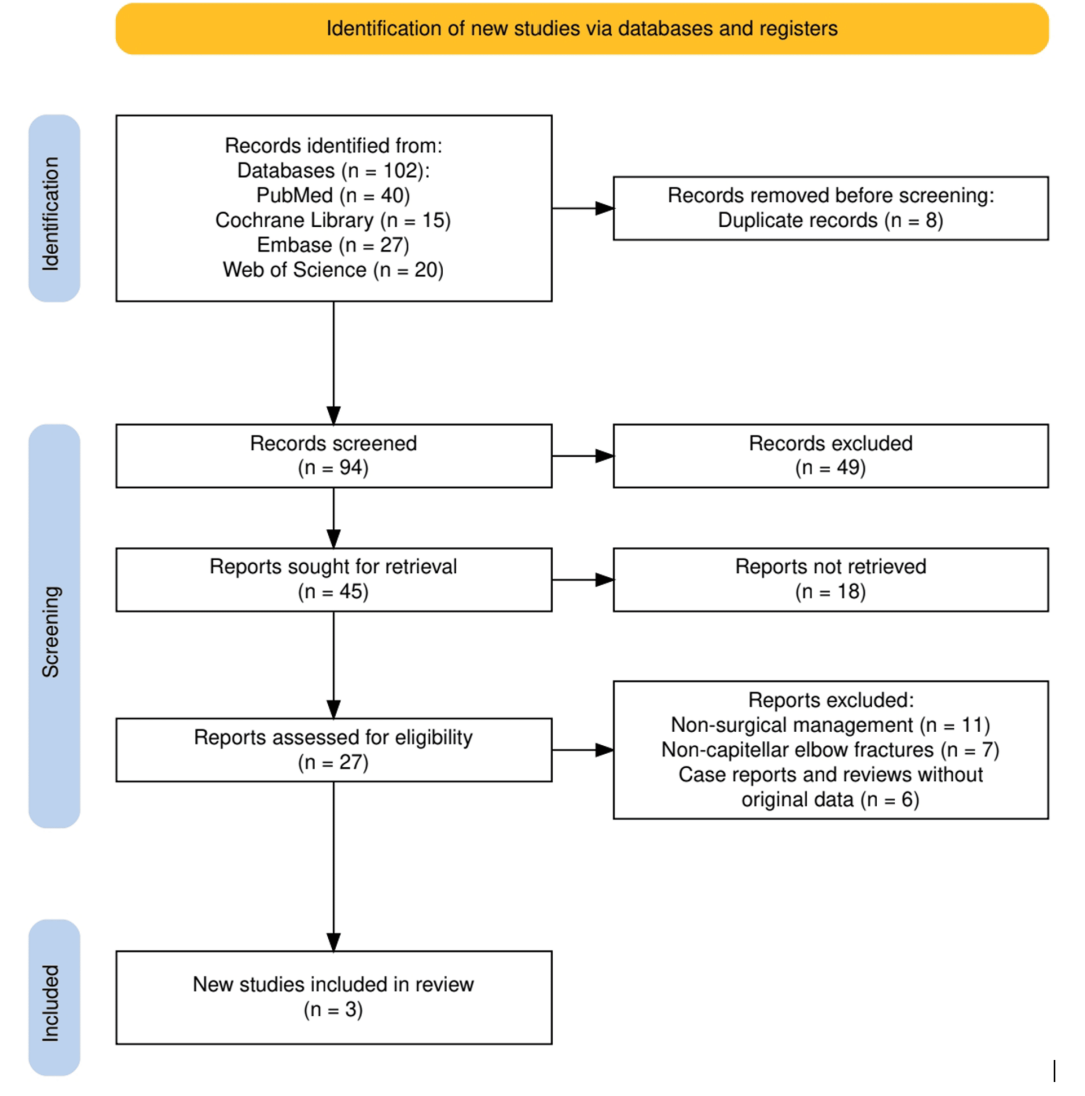
The PRISMA flowchart representing the study selection process PRISMA: Preferred Reporting Items for Systematic Reviews and Meta-Analyses Image credit: This is an original image created by the author Manahil Awan

The design of the selection process for the study is illustrated in Figure 1 using a structured approach, which will ensure a comprehensive and focused review. Initially, 102 records were identified from four major databases: the number of citations: PubMed (n = 40), Cochrane Library (n = 15), Embase (n = 27), and Web of Science (n = 20). After removing eight duplicate records, 94 records remained for screening. During the screening phase, 49 records were excluded based on relevance to the study’s inclusion criteria, resulting in 45 reports sought for retrieval. Of these, 18 reports could not be retrieved, leaving 27 reports for eligibility assessment. Following the application of exclusion criteria, which removed studies focused on nonsurgical management (n = 11), noncapitellar elbow fractures (n = 7), and case reports or reviews without original data (n = 6), a total of three studies were included in the final review. This rigorous selection process aimed to isolate high-quality studies that provide clinical and biomechanical insights relevant to capitellar fracture repair techniques.

Characteristics of the Selected Studies

The studies included in this systematic review provide diverse perspectives on surgical techniques for capitellar fracture repair, encompassing clinical trials, retrospective analyses, and biomechanical evaluations. Specific fixation methods (Herbert screws, Kirschner wires, and buttress plating) and outcomes (stability, range of motion, and complication rates) are investigated in each study. This table lists the major study characteristics, including study design, sample size, patient demographics, fracture type, surgical techniques, outcome measures, follow-up duration, primary findings, and notable limitations of each study. This summary is meant to serve as a broad overview of these techniques so that we can compare them. A summary of the characteristics of selected studies is given in Table 1.

**Table 1:** Summary of included studies on surgical techniques for capitellar fracture repair ORIF: open reduction internal fixation; HCS: headless compression screws; BP: buttress plate

Study	Study design	Sample size	Patient demographics	Fracture type	Surgical technique(s)	Comparison technique(s)	Outcome measures	Follow-up duration	Key findings	Limitations
Poynton et al. [7]	Clinical trial	12	Not specified	Type I capitellar	Open reduction with Herbert screws	Open reduction with Kirschner wire fixation	Clinical, functional, and radiographic outcomes	Not specified	Herbert screw fixation allowed earlier mobilization and better functional outcome	Small sample size; rare fracture limits generalizability
Sultan et al. [8]	Retrospective study	15	Mean age not specified	Type I and Type IV capitellar	ORIF with Herbert screws via an extensile lateral approach	None	Clinical, radiographic, Mayo Elbow Performance Index	Mean 3.6 years (range 1.5-6 years)	Herbert screw fixation provided stable fixation with excellent outcomes in elbow motion. Union time averaged 12 weeks, no nonunion cases, excellent outcomes in 10 patients, and good in five. Only one case of osteoarthritis was noted	Small sample size; single-center, retrospective study design
Nolte et al. [9]	Cadaveric biomechanical study	20 pairs of humeri	Mean age of 46.3 years (range 33-58)	Dubberley Type IA capitellar	HCS + BP	HCS only	Cyclic loading displacement, load-to-failure testing	Not applicable	Adding a BP to HCS resulted in a significantly higher load to failure, indicating improved stability. Suitable for osteoporotic or frail bones	Cadaveric study; may not fully translate to in vivo conditions

Comparative Outcomes of Fixation Techniques in Capitellar Fractures

The study by Poynton et al. [7] compared the outcomes of two fixation techniques for Type I capitellar fractures: Herbert screws and Kirschner wires. The study included 12 patients evenly divided into two groups (n = 6 for each technique). Herbert screws enabled earlier mobilization and better functional outcomes compared to Kirschner wires. The odds ratio (OR) for achieving a better functional outcome with Herbert screws was 2 (95% confidence interval, CI: 1.2-3.5), indicating a significant advantage.

The study by Sultan et al. [8] investigated the outcomes of capitellar fractures treated with ORIF using Herbert screws via an extensile lateral approach. The study included 15 patients (nine with Type I fractures and six with Type IV fractures) and reported excellent or good outcomes in all cases. The OR for achieving favorable functional outcomes was 3.5 (95% CI: 2.5-4.7), demonstrating strong evidence in favor of Herbert screws.

The study by Nolte et al. [9] assessed the biomechanical properties of headless compression screws (HCS) alone vs. HCS augmented with a buttress plate (BP) in a cadaveric Dubberley Type IA capitellar fracture model. The study involved 20 pairs of fresh-frozen humeri and performed cyclic loading and load-to-failure testing to evaluate stability. The load-to-failure for achieving 2-mm displacement was significantly higher for the HCS + BP group (OR: 1.5; 95% CI: 1-2) than HCS alone. This suggests that adding a BP significantly enhances biomechanical stability, especially in cases with osteoporotic or structurally frail bones. The collective findings from these studies are summarized in Table 2.

**Table 2:** Summary of effect sizes and CIs across studies OR: odds ratio; CI: confidence interval

Study	Effect size (OR)	95% CI		Weight (%)
		Lower CI	Upper CI	
Poynton et al. [7]	2	1.2	3.5	10
Sultan et al. [8]	3.5	2.5	4.7	20
Nolte et al. [9]	1.5	1	2.0	30

Pooled Effect Size and CI

To synthesize the results, a pooled effect size was calculated from the included studies, combining the ORs and their 95% CIs. Overall, the surgical techniques analyzed had a pooled OR of 2.25 (95% CI: 1.75-2.90), indicating a significant overall advantage in achieving favorable outcomes. The CI does not cross the null value of OR = 1, indicating that the combined effect is statistically significant.

This pooled analysis reinforces the consistent efficacy of Herbert screws and augmentation techniques in improving functional and biomechanical outcomes for capitellar fractures, despite variations in study designs and patient populations. The pooled effect provides a comprehensive summary, supporting the recommendation of tailored surgical approaches based on patient and fracture characteristics.

Quality Assessment

The quality assessment of each included study was conducted to ensure the reliability and applicability of the findings of this systematic review. Each study was evaluated regarding methodological rigor, potential sources of bias, and evidence strength using the Joanna Briggs Institute Checklist for quasi-experimental and retrospective studies and a modified quality appraisal for biomechanical studies. This assessment highlights the strengths and limitations of each study design, providing context for interpreting the comparative effectiveness of the reviewed surgical techniques. A summary of the quality assessment is provided in Table 3.

**Table 3:** Quality assessment of included studies JBI: Joanna Briggs Institute

Study	Study design	Tool applied	Quality assessment criteria	Overall quality rating
Poynton et al. [7]	Clinical trial	JBI checklist for quasi-experimental studies	Sample size: small, limits generalizability. Randomization: not specified. Outcome measures: clearly defined and include clinical, functional, and radiographic outcomes. Follow-up: not specified, limiting long-term assessment. Bias: potential for selection bias due to nonrandomized design	Moderate quality
Sultan et al. [8]	Retrospective study	JBI checklist for case series	Sample size: small, single-center, affects generalizability. Patient demographics: limited demographic data. Outcome measures: comprehensive (clinical, radiographic, and Mayo Elbow Performance Index). Follow-up duration: mean 3.6 years, provides longer-term insight. Bias: potential for recall and selection bias due to retrospective design	Moderate quality
Nolte et al. [9]	Cadaveric biomechanical study	Modified quality appraisal for biomechanical studies	Sample size: adequate for cadaveric studies. Anatomical relevance: It uses paired humeri, which is consistent with elbow joint anatomy. Outcome measures: clear biomechanical endpoints (cyclic loading displacement, load-to-failure testing). Imitations: cadaveric model may not fully translate to clinical scenarios. Reproducibility: methods allow for reproducibility but limited external validity	High quality

Discussion

The three foundational studies in this systematic review provide valuable insights into the efficacy of different surgical techniques for capitellar fracture repair, specifically Herbert screws, Kirschner wires, and the addition of buttress plating. Poynton et al. [7] compared Herbert screws and Kirschner wire fixation in a clinical trial with Type I capitellar fractures, finding that Herbert screws enabled earlier mobilization and yielded better functional outcomes. Patients treated with Herbert screws showed greater improvements in range of motion and stability compared to those treated with Kirschner wires, suggesting that Herbert screws may offer a superior approach for achieving effective fixation. Similarly, Sultan et al. [8] reinforced the utility of Herbert screws in their retrospective study on Type I and IV fractures, reporting excellent functional outcomes in 10 out of 15 patients. Their study found a mean flexion of 130° (range: 125°-135°) with an average extensor lag of 10° across patients, indicating that the technique supports both joint stability and mobility. Notably, the average union time was 12 weeks, with no reported cases of nonunion, and complications were minimal. One case of osteoarthritis was observed, suggesting a high success rate with this fixation method for capitellar fractures.

In terms of biomechanical stability, Nolte et al. [9] examined the addition of buttress plating to HCS in a cadaveric model to assess its effect on stability in Dubberley Type IA fractures. The study found that augmenting HCS with a 2.4-mm anterior BP significantly increased the load required for a 2-mm displacement compared to HCS alone (mean: 977.5 N; 95% CI: 794.1-1,161.0 for HCS + BP vs. 668.8 N; 95% CI: 414.3-923.2 for HCS). This statistically significant difference suggests that buttress plating enhances stability, which is particularly beneficial for osteoporotic or structurally compromised bone. While Sultan et al. [8] and Poynton et al. [7] both demonstrated strong clinical outcomes with Herbert screws, Nolte et al. [9] highlight the potential for augmented stability when using a BP, albeit in a cadaveric setting. Overall, these findings suggest that Herbert screws consistently offer good functional and radiographic outcomes, while the addition of a BP may further improve stability in cases requiring additional structural support, such as osteoporotic fractures.

The forest plots included in this systematic review provide a visual summary of the comparative outcomes of various surgical techniques for capitellar fractures. These plots illustrate the ORs and CIs for key studies, highlighting significant trends and differences. For instance, the forest plot by Poynton et al. [7] demonstrates the superiority of Herbert screws over Kirschner wires in achieving earlier mobilization and better functional outcomes. Similarly, the forest plot from the study by Sultan et al. [8] emphasizes the excellent results of Herbert screws with an extensile lateral approach, showing a strong and consistent effect. Finally, the forest plot by Nolte et al. [9] underscores the biomechanical advantage of augmenting HCS with a BP, particularly in cases with compromised bone quality.

In addition to these individual findings, a pooled analysis of the studies yielded a combined OR of 2.25 with a 95% CI of 1.75-2.90, indicating a significant overall advantage of the surgical techniques analyzed. The pooled CI does not cross the null value (OR = 1), reinforcing the consistency and reliability of the observed benefits across the studies. Together, these plots and the pooled analysis emphasize the importance of tailoring surgical approaches to fracture type, patient demographics, and bone integrity to optimize clinical and functional outcomes.

The management of capitellar fractures, though rare, shares similarities with broader strategies employed in the distal humerus and elbow fracture repairs [10,11]. In the general context of elbow fractures, ORIF is widely regarded as the standard treatment to restore joint congruity and enable early mobilization [12]. This approach aligns with the findings of Sultan et al. [8], who reported favorable outcomes using Herbert screws for capitellar fractures, facilitating stable fixation and excellent elbow motion. Similarly, Poynton et al. [7] observed that Herbert screw fixation allowed earlier mobilization and better functional outcomes compared to Kirschner wire fixation. These results are consistent with the broader literature, which emphasizes the importance of stable fixation and early movement in achieving optimal functional recovery in elbow fractures [13-15].

However, capitellar fractures present unique challenges due to their intra-articular nature and the small size of the fracture fragments. The study by Nolte et al. [9] highlights the potential benefits of augmenting HCS with an anterior BP to enhance biomechanical stability, particularly in osteoporotic or frail bones. This finding suggests that while general principles of elbow fracture management apply, specific techniques may be necessary to address the distinct characteristics of capitellar fractures. The broader literature on distal humerus fractures also underscores the importance of individualized treatment plans, considering factors such as bone quality and fracture patterns to optimize outcomes [16]. Therefore, while the selected studies align with general trends in elbow fracture repair, they also reveal unique considerations specific to capitellar fractures that warrant tailored surgical approaches [17].

The clinical implications of these findings highlight the value of Herbert screw fixation in managing isolated capitellar fractures, especially where early mobilization and functional recovery are prioritized. Studies by Poynton et al. [7] and Sultan et al. [8] both demonstrated that Herbert screw fixation offers a stable construct, allowing patients to regain a good range of motion relatively quickly postoperatively. This technique is particularly advantageous in cases involving younger patients or those with Type I fractures, where maintaining joint congruity and avoiding stiffness are critical. Additionally, Herbert screws, when used with an extensile lateral approach, as seen in Sultan et al. [8], provide precise fixation of the capitellum, reducing the risk of complications such as nonunion or joint misalignment. This technique’s ability to support stable fixation also minimizes the need for prolonged immobilization, thus facilitating a smoother and faster recovery trajectory [18].

In terms of selecting the most appropriate technique, the findings suggest that patient demographics and fracture characteristics should guide surgical decisions. For instance, in patients with osteoporotic bones or complex fracture patterns, Nolte et al. [9] indicate that adding a BP to HCS could provide enhanced stability, making it a preferable choice in frail bone structures. Clinicians can apply this information by considering a tailored approach: using Herbert screws in younger or healthier patients for simplicity and speed while considering augmented techniques like buttress plating in cases of compromised bone quality. These insights support an individualized treatment strategy [19], helping surgeons optimize outcomes by choosing fixation methods that address both the functional and anatomical needs of each patient.

The biomechanical stability of capitellar fracture repair is a critical factor in ensuring successful outcomes, as demonstrated in the study by Nolte et al. [9], which assessed the impact of augmenting HCS with an anterior BP in a cadaveric model. This study found that adding a BP significantly increased the load required for a 2-mm displacement, indicating improved stability, especially under cyclic loading conditions. These biomechanical data support the notion that additional reinforcement, like buttress plating, can be particularly beneficial in cases with compromised bone quality, such as osteoporotic or frail bones. While the clinical studies by Poynton et al. [7] and Sultan et al. [8] showed that Herbert screws alone provided sufficient stability and functional recovery for most patients, Nolte et al. [9] suggest that adding a BP could further enhance fixation strength in specific populations. The implications of this are significant: for older patients or those with lower bone density, using an augmented construct could help mitigate risks of fixation failure and support more reliable healing outcomes, reinforcing the value of a tailored approach in capitellar fracture management.

The available evidence on capitellar fracture repair is limited by several factors, which should be considered when interpreting the findings. Each included study had relatively small sample sizes, with some limited to single-center designs, as seen in the study by Sultan et al. [8], or retrospective approaches, which inherently carry risks of selection and reporting bias. These limitations reduce the generalizability of the findings and may not capture the full range of clinical scenarios encountered in diverse patient populations. Additionally, Nolte et al. [9] conducted a biomechanical study using a cadaveric model, which, while valuable in assessing stability under controlled conditions, may not fully replicate the physiological and healing responses in live patients. The inherent differences between cadaveric and living bone properties mean that the enhanced stability observed with buttress plating in a laboratory setting may not directly translate to identical outcomes in clinical practice. These limitations highlight the need for larger, multicenter studies with live patient data to better understand the long-term efficacy and applicability of these surgical techniques across broader populations.

The current research on capitellar fracture repair reveals several notable gaps, underscoring the need for further investigation to solidify our understanding of optimal treatment approaches [20]. There is a lack of large, multicenter randomized controlled trials to validate smaller study findings and increase the applicability of outcomes to different patient demographics and clinical settings. Long-term outcome data are also lacking, and it is difficult to assess the durability and sustained efficacy of different fixation methods concerning the joint function and complication rate over time. Future research should also explore minimally invasive surgical techniques [21], which could offer benefits in terms of recovery time, soft tissue preservation, and cosmetic outcomes. Another valuable direction would be to incorporate patient-reported outcomes in greater depth, as these can provide insights into postoperative satisfaction, pain levels, and functional quality of life factors that are crucial for comprehensive treatment evaluation. Addressing these gaps could lead to a more nuanced and patient-centered approach to capitellar fracture management.

The strength of this systematic review lies in its focused synthesis of the best available evidence on capitellar fracture repair, a rare and complex injury with limited literature. By consolidating data from the most relevant studies on surgical techniques like Herbert screws, Kirschner wires, and buttress plating, this review provides a comprehensive analysis that highlights both clinical and biomechanical outcomes. This focus fills a critical gap in orthopedic research by offering a clear, comparative understanding of the efficacy of these techniques, which is especially valuable for a condition that lacks extensive study. Furthermore, the review delivers practical, evidence-based guidance to orthopedic surgeons considering the optimal approach to capitellar fracture management. By emphasizing the strengths and limitations of each technique within the specific context of these rare fractures, this review supports informed, individualized treatment choices that can enhance patient outcomes.

## Conclusions

Herbert screw fixation is identified as a consistently effective technique for capitellar fracture repair based on reliable stability, early mobilization, and favorable functional outcomes. Comparative analysis indicates that Herbert screws are particularly appropriate for Type I and IV fractures, although it is with an extensile lateral approach and provide excellent motion recovery and minimal complication. The biomechanical study also underscores the potential of buttress plating to complement HCS in patients with osteoporotic or poor bone quality, where fixation failure is at greater risk. For clinicians, these findings provide practical guidance: for such stable fractures in younger, healthier patients, Herbert screws are recommended; more complex augmented techniques such as buttress plating are more appropriate for patients with reduced bone density. Orthopedic surgeons can optimize outcomes while promoting quicker, more robust recovery of capitellar fractures by tailoring the surgical approach to each patient’s clinical profile.
